# Case report of an unusual hepatic abscess caused by *Actinomyces odontolyticus* in a patient with human immunodeficiency virus infection

**DOI:** 10.1186/s12879-021-06703-6

**Published:** 2021-09-23

**Authors:** Shao-Lun Hsu, Chin-Ting Wu, Yuan-Chen Chang, Chia-Kwung Fan, Yuarn-Jang Lee

**Affiliations:** 1 Medical Education Department, Taipei Medical University Hospital, Taipei, Taiwan; 2 Department of Molecular Parasitology and Tropical Diseases, School of Medicine, College of Medicine, Taipei Medical University, Taipei, Taiwan; 3 School of Medicine, College of Medicine, Taipei Medical University, 250 Wuxing Street, Xinyi Dist., Taipei City, 11031 Taiwan; 4 Center for Infection Control, National Taiwan University Hospital, Taipei, Taiwan; 5 Medical Education Department, Chang Gung Memorial Hospital, Linkou Medical Center, Taoyuan, Taiwan; 6 Division of Infectious Diseases, Department of Internal Medicine, Taipei Medical University Hospital, 252 Wuxing Street, Xinyi Dist., Taipei City, 11031 Taiwan

**Keywords:** *Actinomyces odontolyticus*, *Streptococcus constellatus*, *Candida albicans*, Liver abscess, Human immunodeficiency virus

## Abstract

**Background:**

*Actinomyces odontolyticus* is not commonly recognized as a causative microbe of liver abscess. The detection and identification of *A. odontolyticus* in laboratories and its recognition as a pathogen in clinical settings can be challenging. However, in the past decades, knowledge on the clinical relevance of *A. odontolyticus* is gradually increasing. *A. odontolyticus* is the dominant oropharyngeal flora observed during infancy [Li et al. in Biomed Res Int 2018:3820215, 2018]. Herein we report a case of severe infection caused by *A. odontolyticus* in an immunocompromised patient with disruption of the gastrointestinal (GI) mucosa.

**Case presentation:**

We present a unique case of a patient with human immunodeficiency virus infection who was admitted due to liver abscess and was subsequently diagnosed as having coinfection of *A. odontolyticus*, *Streptococcus constellatus*, and *Candida albicans* during the hospital course. The empirical antibiotics metronidazole and ceftriaxone were replaced with the intravenous administration of fluconazole and ampicillin. However, the patient’s condition deteriorated, and he died 3 weeks later.

**Conclusion:**

This report is one of the first to highlight GI tract perforation and its clinical relevance with *A. odontolyticus* infection. *A. odontolyticus* infection should be diagnosed early in high-risk patients, and increased attention should be paid to commensal flora infection in immunocompromised individuals.

## Background

*Actinomyces* species are nonmotile, filamentous, anaerobic, Gram-positive, rod-shaped bacteria that are commensal flora in the dental plaque and nasopharyngeal and gastrointestinal (GI) tracts [1]. *Actinomyces* infection is rare and generally viewed as an opportunistic infection because it predominantly affects immunosuppressed patients. Although the prognosis of *Actinomyces* infection is usually favorable under medical treatment, it can still lead to death in patients with extensive involvement or those without early detection. Among 25 *Actinomyces* species identified in the human microbiota, *A. gerencseriae* and *A. israelii* are the most commonly reported species in human diseases and are responsible for approximately 50% of cervicofacial actinomycotic infections [2, 3]. Isolation of *A. odontolyticus* from liver abscess is exceptionally rare, and early diagnosis of *A. odontolyticus* infection is considerably challenging in clinical management. Herein we present the case of a human immunodeficiency virus (HIV)-infected patient with esophageal corrosive injury who developed liver abscess caused by *A. odontolyticus* infection. This article is one of the first to review the limited literature on *A. odontolyticus* infection and highlight its clinical relevance with previous GI tract perforation.

## Case presentation

A 45-year-old male, HIV-positive patient who was receiving regular antiretroviral therapy and had an undetectable viral load and a CD4 count of 358.31 cells/µL presented to the emergency department with a 2-week history of fever and chills.

Two years ago, the patient developed an esophageal corrosive injury caused by drinking a strong alkali in an attempt to commit suicide. He was discharged from the hospital after receiving intensive care. Six months later, the patient developed burning and foreign body sensation in the throat. Balloon dilatation was performed due to the detection of an esophageal stricture through esophagoscopy. However, after 2 months, the patient complained of recurrent dysphagia, because of which he was dependent only on a full liquid diet. A severe esophageal stricture was detected through repeated esophagoscopy; instead of esophageal reconstruction or gastrostomy, jejunostomy was performed considering erosive injury in both the esophagus and stomach. In the following 6 months, he experienced an episode of esophagitis and lost 20 kg of his body weight.

Two weeks before this admission, he complained of fever and chills with dizziness, vomiting, and fatigue. On examination in the emergency room, his body weight was 40 kg, and his BMI was 14. Vital sign assessment revealed a body temperature of 38.7 °C, a pulse rate of 139 bpm, a respiratory rate of 18 breaths per minute, a blood pressure of 95/67 mmHg, and oxygen saturation of 98%. Tenderness over the right upper quadrant of the abdomen was noted.

Leukocytosis with a WBC count of 12.81 × 10^3^/μL (reference range: 4 × 10^3^/μL to 11 × 10^3^/μL) with a left shift and an elevated C-reactive protein level of 22.68 mg/dL (reference range < 0.5 mg/dL) were observed. The hemoglobin level was 6.9 g/dL (reference range 13.0–17.0 g/dL). The platelet count was 634 × 10^3^/μL (reference range 130–400 × 10^3^/μL) with a prolonged prothrombin time. The prothrombin time/international normalized ratio was 1.55 (reference range 0.78–1.12), and activated partial thromboplastin time was 46.0 s (reference range 32.0–45.1 s). Liver function test levels were elevated, with the glutamic oxaloacetic transaminase level being up to 129 U/L (reference range < 40 U/L). Hypoalbuminemia was noted, with the albumin level being 2.2 g/dL (reference range: 3.5–5.2 g/dL). The lactate level was 19.9 mg/dL (reference range: 4.5–19.8 mg/dL). The CD4+ count was 358.31 cells/µL (reference range: 404.00–1612.00 cells/µL).

**Fig. 1:**
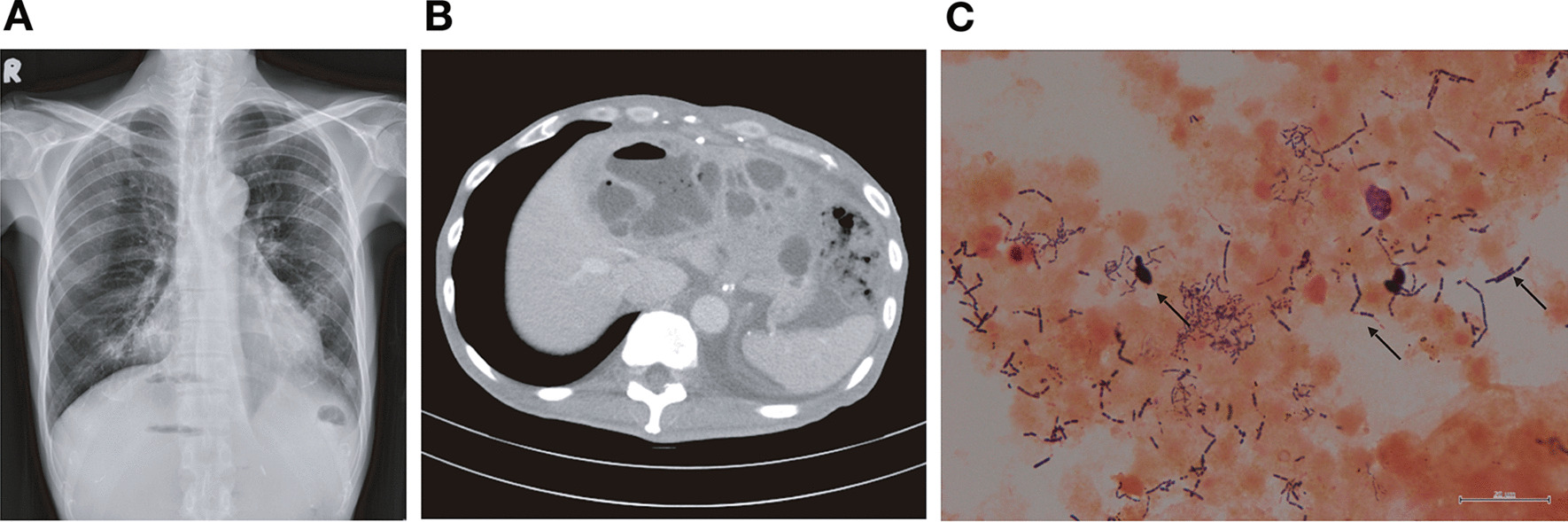
Clinical image and laboratory findings suggest a polymicrobial infection in liver abscess from an HIV-infected patient. **A** Chest X-ray obtained on the patient’s first hospitalization day revealed an air-fluid level beneath the right diaphragm, and **B** contrast-enhanced computed tomography of the abdomen demonstrated a 14 × 7.6-cm multiloculated cystic lesion at the level of S2, S3, and S4 in the liver parenchyma. The lesion presented with an air-fluid level and multiple enhancing septa. **C** Gram staining of the specimen aspirated from liver abscess revealed the budding yeast, long-chain Gram-positive coccus, and Gram-positive bacilli without branching (from left to right by the arrows)

Chest X-ray revealed an air-fluid level beneath the right diaphragm (Fig. 1A). Abdominal computed tomography (Fig. 1B) demonstrated a multiloculated cystic lesion occupying the entire left lobe of the liver. Contrast collection within the gastric tube and esophagus revealed leakage of contrast medium from the distal esophagus suture that was possibly connected with liver abscess. A total of 160 mL of the purulent exudate was drained from liver cysts. Under the impression of liver abscess with impending septic shock, an intravenous fluid challenge with an inotropic agent and the empirical antibiotics metronidazole and ceftriaxone were administered immediately.

*Candida albicans*, *Streptococcus constellatus*, and *A. odontolyticus* were isolated (Fig. 1C), and their presence was confirmed through matrix assisted laser desorption ionization-time of flight (MALDI-TOF) mass spectrometry 7 days later. The presence of *A. odontolyticus* was reconfirmed based on the negative results of oxidase and catalase tests. Metronidazole and ceftriaxone were replaced with the intravenous administration of fluconazole and ampicillin. However, the patient’s condition deteriorated, and he died 3 weeks later.

## Discussion and conclusion

*Actinomyces* species are facultatively anaerobic, Gram-positive, rod-shaped bacteria that are the commensal flora of the oral cavity, nasopharyngeal tract, GI tract, and skin [1]. They mostly affect immunodeficient or immunocompromised individuals who have other underlying diseases [1]. Once the anatomical barrier is disrupted, *Actinomyces* accompanied with other pathogenic bacteria in the alimentary tract may invade into adjacent tissues and cause infection [1, 3]. Actinomycosis lesions in humans usually consist of other aerobic or anaerobic species such as *A. actinomycetemcomitans*, *Eikenella corrodens*, *Capnocytophaga*, *Fusobacteria*, *Bacteroides*, *Staphylococci*, *Streptococci*, and *Enterobacteriaceae* [4].

In the present case, corrosive injury throughout the alimentary tract and jejunostomy were considered potential predisposing factors contributing to infection caused by oropharyngeal flora including *C. albicans*, *S. constellatus*, and *A. odontolyticus*.

Valour et al. [3] reported that *A. gerencseriae and A. israelii* are the dominant species isolated from 70% of human forms of actinomycosis. Isolation of *A. odontolyticus* from liver abscess is exceptionally rare. Moreover, *Actinomyces* species such as *A. odontolyticus*, *A. meyeri*, and *A. graevenitzii* have been reported to be isolated from the oral cavity (including the tongue surface, tonsillar crypts, and distal esophagus [1]) in one-third of infants at the age of 2 months, and the diversity of *Actinomyces* increases with age [5]. In particular, *A. odontolyticus* is the predominant species in the oral cavity of edentulous infants and is the only representative of the genus found at an early age [5, 6].

In our patient, the repair and regeneration of the oral mucosa were similar to those observed in edentulous infants, which may be the reason for the unusual infection caused by *A. odontolyticus* instead of other *Actinomyces* species.

*Actinomyces odontolyticus* resides on mucosal surfaces and gains access into the deeper tissue through trauma [1]. Previous esophageal corrosive injury as well as hypoimmunity may have contributed to the severe infection in our patient. Few reports of *A. odontolyticus* infection shared similar characteristics with our case.

In their case report, Považan et al. described *A. odontolyticus*-associated bacteremia in a patient on the 10th hospital day after chemotherapy treatment [7]. A study by Cone et al. summarized 25 cases of *A. odontolyticus* infection from 1974 to 2003, and five of the infected patients were immunosuppressed [8]. Lopes et al. described peritonitis caused by *A. odontolyticus* in a patient with underlying gastric perforation [9]. Furthermore, Deivert et al. reported hepatic actinomycosis in an immunocompetent patient who had undergone endoscopic and surgical treatment due to hepatic artery pseudoaneurysm. They presumed that previous interventions provided a portal of entry for bacteria, leading to indolent abscess formation [10].

The application of prolonged therapies with high doses of amoxicillin or penicillin G remains the first-line treatment against *Actinomyces*. However, some studies have suggested tetracyclines or doxycycline as alternative regimens [11, 12]. Abscess drainage was performed in patients with widespread necrotic tissues or those nonresponsive to antibiotic therapy. In some cases, blood supply to infected sites was insufficient for antibiotics to penetrate; consequently, surgery was considered [11]. However, aggressive treatment was not favored in our patient considering his asthenia condition and personal reasons.

In summary, actinomycosis is still a disease that poses a considerable diagnostic challenge in clinical settings. Coinfection of oropharyngeal flora including *C. albicans*, *S. constellatus*, and *A. odontolyticus* is rarely reported. In the present case, a history of GI tract perforation provided crucial clues toward unidentified bacterial diseases among immunocompromised individuals. With the increasing availability of molecular identification techniques, such as MALDI-TOF, the reported number of cases of *A. odontolyticus* infection is likely to increase. In conclusion, *A. odontolyticus* should be considered as a causative pathogen in patients with disruption of the GI mucosa. We emphasize the importance of early diagnosis, and delays in treatment can lead to increased morbidity and mortality.

## Data Availability

The datasets used during the current study are available from the corresponding author on reasonable request.
